# Translational Attenuation by an Intron Retention in the 5′ UTR of *ENAM* Causes Amelogenesis Imperfecta

**DOI:** 10.3390/biomedicines9050456

**Published:** 2021-04-22

**Authors:** Youn Jung Kim, Yejin Lee, Hong Zhang, John Timothy Wright, James P. Simmer, Jan C.-C. Hu, Jung-Wook Kim

**Affiliations:** 1 Department of Molecular Genetics & DRI, School of Dentistry, Seoul National University, Seoul 03080, Korea; ykim71@snu.ac.kr; 2 Department of Pediatric Dentistry & DRI, School of Dentistry, Seoul National University, Seoul 03080, Korea; lyj72255621@gmail.com; 3 Department of Biologic and Materials Sciences, School of Dentistry, University of Michigan, Ann Arbor, MI 48108, USA; zhanghon@umich.edu (H.Z.); jsimmer@umich.edu (J.P.S.); 4 Division of Pediatric & Public Health Dentistry, School of Dentistry, University of North Carolina, Chapel Hill, NC 27514, USA; tim_wright@unc.edu

**Keywords:** whole exome sequencing, *ENAM*, amelogenesis imperfecta, hereditary enamel defects, intron retention, splicing donor site mutation

## Abstract

Amelogenesis imperfecta (AI) is a collection of rare genetic conditions affecting tooth enamel. The affected enamel can be of insufficient quantity and/or altered quality, impacting structural content, surface integrity and coloration. Heterozygous mutations in *ENAM* result in hypoplastic AI without other syndromic phenotypes, with variable expressivity and reduced penetrance, unlike other AI-associated genes. In this study, we recruited a Caucasian family with hypoplastic AI. Mutational analysis (using whole exome sequencing) revealed a splicing donor site mutation (NM_031889.3: c. −61 + 1G > A). Mutational effects caused by this variant were investigated with a minigene splicing assay and in vitro expression analysis. The mutation resulted in a retention of intron 1 and exon 2 (a normally skipped exon), and this elongated 5′ UTR sequence attenuated the translation from the mutant mRNA. Structure and translation predictions raised the possibility that the long complex structures—especially a hairpin structure located right before the translation initiation codon of the mutant mRNA—caused reduced protein expression. However, there could be additional contributing factors, including additional uORFs. For the first time, we determined that a mutation altered the *ENAM* 5′ UTR, but maintained the normal coding amino acid sequence, causing hypoplastic AI.

## 1. Introduction

Tooth enamel is the hardest tissue in the human body. It covers the crown of the tooth and provides a strong protective barrier to withstand masticatory forces. The formation of tooth enamel (amelogenesis) begins with the secretion of the enamel matrix from differentiated enamel forming cells (ameloblasts). The developing enamel matrix facilitates the formation of long, ribbonlike enamel crystallites during the secretory stage, but undergoes degradation and removal when ameloblasts transition into the maturation stage. Matured enamel has a unique structure comprised of exceptionally compact hexagonal enamel crystals containing 95% mineral, 1% organic materials, and 4% water by weight [1].

Hereditary defects involving tooth enamel, such as amelogenesis imperfecta (AI), can be classified according to clinical phenotype into three (hypoplastic, hypocalcification, and hypomaturation) or two (hypoplastic and hypomineralization) categories [2]. To date, more than 20 genes are known to cause AI [3]. Among them, hypoplastic nonsyndromic AI is caused by mutations in genes encoding amelogenin (*AMELX*) in an X-linked inheritance [4], enamelin (*ENAM*) [5] and laminin beta-3 (*LAMB3*) [6] in an autosomal dominant inheritance, and ameloblastin (*AMBN*) [7] and acid phosphatase 4 (*ACP4*) [8] in an autosomal recessive inheritance.

AI is largely fully penetrant, and expressivity is not highly variable. An earlier epidemiologic study of a Swedish population raised the possibility of hypoplastic AI with incomplete penetrance [9]. Although there have been several reports of variable expression in families with *LAMB3* or *ENAM* mutations [10,11,12], to the best of our knowledge, *ENAM* is the only gene reported to have a lack of penetrance to date [13,14].

In this study, we recruited a five-generation Caucasian family with hypoplastic AI. Whole exome sequencing analysis identified a pathogenic *ENAM* mutation—a splicing donor site mutation in intron 1. In this study, we reported a 5′ untranslated region (UTR) mutation in *ENAM* for the first time. The mutation effect was characterized by minigene splicing assay and protein expression analysis.

## 2. Materials and Methods

### 2.1. Study Subject Enrollment

The study protocol was independently reviewed and approved by the institutional review board of the Seoul National University Dental Hospital, the University of North Carolina, and the University of Michigan. Informed consent was obtained from participating family members or a guardian. Clinical examinations were performed, and blood samples were collected.

### 2.2. DNA Isolation & Whole Exome Sequencing

Genomic DNA was isolated, and the quality and quantity were measured as described previously [15]. A total of 2.5 μg of DNA samples (III:17 and IV:4) were submitted to Johns Hopkins University Center for Inherited Disease Research (CIDR, Baltimore, MD, USA), and the Agilent SureSelect Human All Exon Enrichment System was used for exome capture. The 125-bp paired-end sequencing reads were generated using Illumina HiSeq 2500 (Illumina, Inc., San Diego, CA, USA).

### 2.3. Bioinformatics

The obtained sequence reads were trimmed to remove adapter sequences and aligned to the reference human genome assembly (hg38). Cutadapt and Burrows–Wheeler Aligner were used for the trimming and alignment, respectively [16,17]. A list of sequence variants was obtained using a series of bioinformatics analysis programs, including Samtools and Genome Analysis Tool Kit [18,19]. Annovar was used to annotate sequence variants with dbSNP build 147 [20]. A minor allele frequency of 0.01 was applied as a cutoff value to filter the variants.

### 2.4. Sanger Sequencing

The identified mutation in the proband was confirmed by Sanger sequencing with the following primers (583 bp, sense: 5′-GCCAAATTCACCAAGGGAAG-3′; antisense: 5′-CAACCAACAAGAAGGAATGCA-3′). Sanger sequencing for all four participating family members was performed at Eurofins Genomics (Louisville, KY, USA).

### 2.5. Cloning and Splicing Assay of the Wild-Type and Mutant ENAM Splicing Vectors

A genomic fragment that encompassed exon 1 to exon 3 of *ENAM* was amplified with primers (1251 bp, sense: 5′-GAGACTTGACTTGACAGCTCCTAT-3′; antisense: 5′-GGATGACTGAGATCCCTTCC-3′) using Pfu Plus 5× PCR Master Mix (Elpis Biotech, Daejeon, Korea). The amplification product was cloned into the pTOP Blunt V2 cloning vector (Enzynomics, Seoul, Korea), and the mutation was introduced by conventional PCR mutagenesis with primers (sense: 5′-CTAATTGGCATTGG**A**TGAGTATTAGAG-3′; antisense: 5′-CTCTAATACTCATCC**A**ATGCCAATTAG-3′). The wild-type and mutant clones were selected and confirmed by Sanger sequencing of the plasmids, and subcloned into a mammalian expression vector (pEGFP-N1) after double digestion with BamHI and XhoI restriction endonucleases. HEK293T cells were transfected with the wild-type and mutant pEGFP-N1 vectors for splicing assay. Total RNA was isolated after 48 h using the RNeasy Plus Mini Kit (Qiagen, Germantown, MD, USA), and cDNA was synthesized with the RTase master mix (Elpis biotech). PCR amplification was performed to check the splicing product(s) with primers (sense: 5′-TTTGAGCCTTTTTGATACTGAACA-3′; antisense: 5′-GTTATCTAGTTTAGGAAAAGAGGTTCC-3′). The amplification bands were excised from an agarose gel and characterized by sequencing.

### 2.6. Western Blot and RT-PCR of the Wild-Type and Mutant ENAM

After confirming the effect of the mutation on the pre-mRNA splicing, additional modification of the cloned vectors was needed to further check the mutational effect on the protein expression. ENAM’s signal peptide (1–39 amino acids) is encoded by exons 3 and 4; therefore, the exon 4 sequence should be added to the vector, and a part of the intron 3 sequence should be removed due to its long length (2115 bp). Mutagenesis primers were designed and used to add the exon 4 sequence between the exon 3 and EGFP coding sequences without intervening nucleotides (e.g., a part of intron 3 and multiple cloning sequences) (sense: 5′-CCTAAACTAGATAACTTGGTACCAAAAGGCAAAATG AAGATTCTCCTGGTCTTTCTAGGGCTTCTTGGTAATTCTGTTGCTATGCCAATGGT GAGCAAGGGC-3′; antisense: 5′-GCCCTTGCTCACCATTGGCATAGCAACAGAATTA CCAAGAAGCCCTAGAAAGACCAGGAGAATCTTCATTTTGCCTTTTGGTACCAAG TTATCTAGTTTAGG-3′; exon 4 sequence is underlined). HEK293 cells were transfected with vectors expressing the wild-type and mutant ENAM tagged with EGFP. After incubation for 30 h, cell lysate and culture media without serum were harvested. Culture media were concentrated using an Amicon ultra-4 centrifugal filter unit (Millipore, Bedford, MA, USA). Rabbit polyclonal anti-GFP primary antibody (AbFrontier, Seoul, Korea) was used at a titer of 1:10,000 and incubated at 4 °C overnight. Goat anti-rabbit IgG secondary antibody, conjugated with HRP (Thermo Fisher Scientific, Rockford, IL, USA), was used at a titer of 1:10,000. The band intensities in the culture media were measured using ImageJ (https://imagej.nih.gov/ij/ (accessed on 1 February 2021)). Mouse monoclonal anti-GAPDH primary antibody (ABM, Richmond, ON, Canada) was used at a titer of 1:20,000 and incubated at 4 ℃ overnight, and goat anti-mouse IgG secondary antibody conjugated with HRP (Thermo Fisher Scientific) was used at a titer of 1:10,000. Silver staining was performed to normalize the amount of culture media. Total RNA was isolated and cDNA was synthesized as described. PCR amplification was performed to assess the expression of the wild-type and mutant ENAM (115 bp, sense: 5′-CATGGTCCTGCTGGAGTTCGTG-3′; antisense: 5′-CCTCTACAAATGTGGTATGG-3′) and GAPDH (309 bp, sense: 5′-CCAAGGTCATCCATGACAAC-3′; antisense: 5′-GCTTCACCACCTTCTTGATG-3′).

### 2.7. Prediction of Secondary Structures and Translation of the Wild-Type and Mutant ENAM mRNA

The wild-type and mutant mRNA sequences encoding ENAM tagged with EGFP were submitted to the RNAfold WebServer (http://rna.tbi.univie.ac.at/cgi-bin/RNAWebSuite/RNAfold.cgi (accessed on 16 February 2021)). Translation in all three frames was analyzed using the Expasy translate tool (https://web.expasy.org/translate/ (accessed on 16 February 2021)) to detect upstream open reading frames (uORFs) and upstream start codons (uAUGs).

## 3. Results

**Figure 1:**
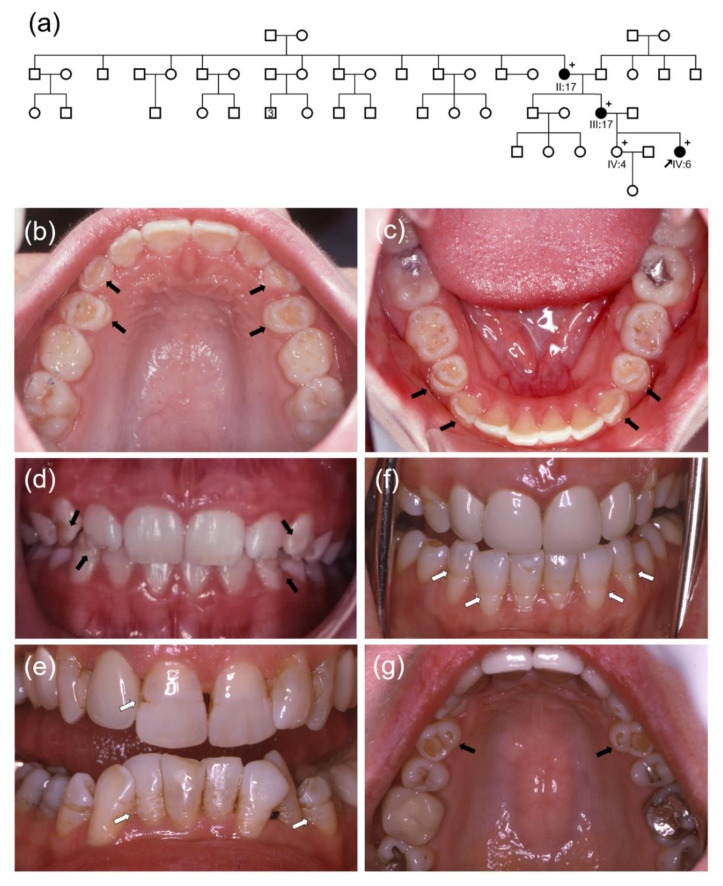

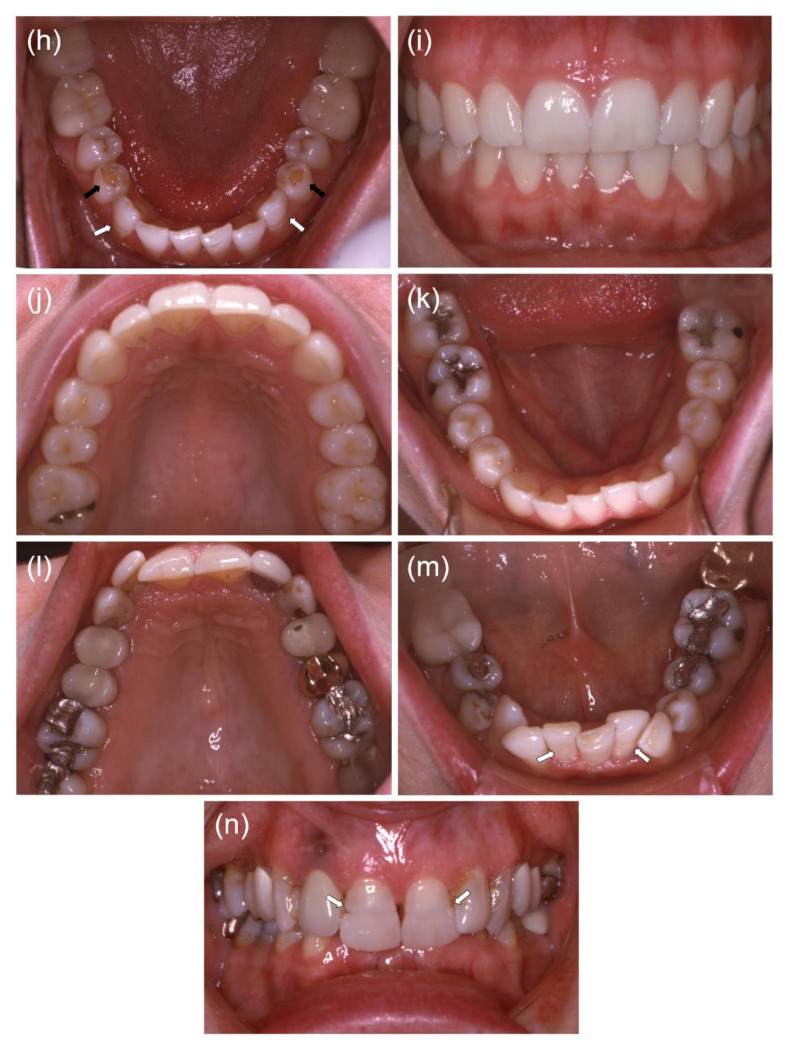
(**a**) Pedigree of the study family. The black arrow denotes the proband. The plus sign (+) indicates participating individuals in this study. The number in the symbol indicates the number of siblings. (**b**–**d**) Clinical photos of the proband (IV:6). Black arrows indicate hypoplastic enamel defects. The maxillary central incisors look normal, and the mandibular incisors do not show horizontal hypoplastic defects. (**e**) Frontal clinical photo of the grandmother of the proband (II:17). White arrows indicate hypoplastic horizontal grooves. (**f**,**g**) Clinical photos of the mother of the proband (III:17). White arrows indicate hypoplastic horizontal grooves. Her maxillary anterior teeth were treated with porcelain full crown prosthetics. Hypoplastic defects in the maxillary first premolars are indicated with black arrows. (**h**) Clinical photos of the mother of the proband (III:17). Hypoplastic defects (black arrows) are shown in the mandibular first premolars, and horizontal hypoplastic grooves (white arrows) are shown in the anterior teeth. (**i**–**k**) Clinical photos of the sister of the proband (IV:4). Her dentition looks normal, without any noticeable hypoplastic enamel defects. (**l**–**n**) Clinical photos of the grandmother of the proband (II:17). White arrows indicate hypoplastic horizontal grooves in the anterior teeth.

The proband was a second daughter (IV:6) in a nonconsanguineous five-generation Caucasian family (Figure 1). She had no remarkable past medical history, but hypoplastic enamel defects were evident on the permanent canines and first premolars. Maxillary lateral incisors also exhibited hypoplastic enamel defects in the distal incisal area. No noticeable hypoplastic enamel defects were evident in the other teeth, including maxillary central and mandibular incisors. Her older sister (IV:4) had normal dentition without enamel defects. Her mother (III:17) and grandmother (II:17) had similar defects, but they also exhibited horizontal hypoplastic grooves in the anterior teeth, a characteristic feature observed in some individuals with *ENAM* mutations.

Exome sequencing was performed with DNA samples from an affected individual and a seemingly unaffected individual (III:17 and IV:4) (Table S1). Data searching to find the filtered variants that were in the affected individual but not in the unaffected individual did not result in a variant in known AI-causing genes. However, a variant in the *ENAM* gene (and no other variant in AI-causing genes) was identified in both individuals.

The identified mutation (NM_031889.3: c. –61 + 1G > A) was a splicing donor site variant in intron 1 of *ENAM*. In silico analysis (NetGene2: http://www.cbs.dtu.dk/services/NetGene2 (accessed on 11 January 2021) and NNSplice: http://www.fruitfly.org/seq_tools/splice.html (accessed on 11 January 2021)) predicted this variant to cause a disruption of the normal splicing donor site. Moreover, the variant was not listed in searched public sequence databases, such as the Exome Variant Server (http://evs.gs.washington.edu/EVS (accessed on 11 January 2021)) and the GnomAD repository (https://gnomad.broadinstitute.org/ (accessed on 11 January 2021)). Sanger sequencing confirmed the mutation in all four participating family members (Figure S1). According to the American College of Medical Genetics and Genomics (ACMG) guideline [21], this variant could be classified as pathogenic with incomplete penetrance in an individual (IV:4).

**Figure 2:**
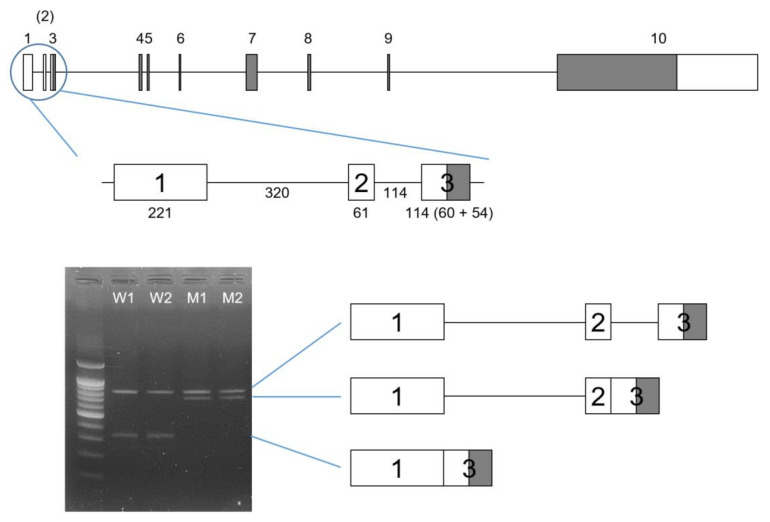
Gene structure diagram of *ENAM*. The boxes indicate the exons, and the lines connecting boxes are the introns. Grey areas in the boxes indicate coding regions, and white areas in the boxes indicate noncoding regions. The exon numbers are shown above the boxes, and the skipped exon 2 is shown in parentheses. Nucleotide numbers are shown below the boxes and the lines in the enlarged diagram that show exons 1 to 3. Exon 3 has 60 noncoding and 54 coding nucleotides. The agarose gel image shows the result of the splicing assay. Wild-type (W1 and W2) vectors resulted in two bands (800 bp upper and 324 bp lower band), while mutant (M1 and M2) vectors resulted in two differently-sized bands (800 bp upper and 705 bp lower band). The common 800 bp upper band was the pre-mRNA sequence. The 324 bp small band was the normal splicing transcript with exon 1 and 3. The 705 bp band was the mutant transcript, including exon 1, intron 1, exon 2 and exon 3. W: wild-type, M: mutant.

The in vitro splicing assay confirmed that the mutation abolished the original splicing donor site and caused retention of a genomic fragment (381 bp) from the 5′ UTR (Figure 2). The mutation resulted in the use of another splicing donor site, which belonged to the normally skipped exon 2. Therefore, the retained fragment included intron 1 (320 bp) and the skipped exon 2 (61 bp). In contrast, the wild-type vector resulted in the inclusion of exons 1 and 3 without exon 2, as in the mRNA sequence (NM_031889.3).

**Figure 3:**
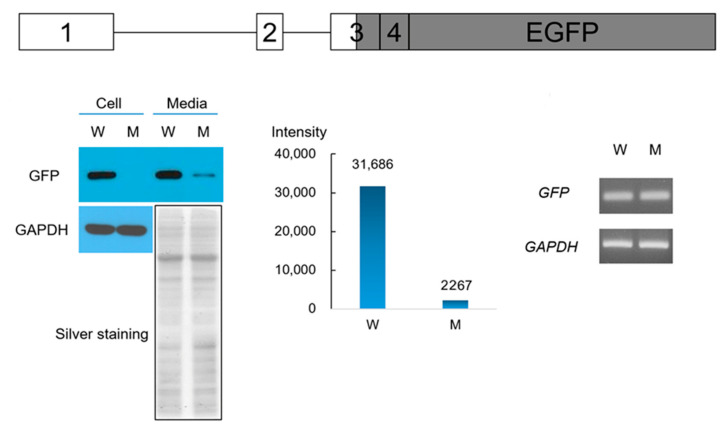
Western blot and RT-PCR of gene expression assay. HEK293 cells were transfected with the wild-type and mutant proteins tagged with C-terminal GFP expressing vectors. A diagram of the gene fragment in the vector is shown above. Coding exons and EGFP encoding sequences are shown with grey color in the boxes. Western blot (using GFP antibody) was performed with the cell lysate and concentrated culture media. There was no detectable protein in the cell lysate transfected with the mutant vector; however, there was a reduced expression in the culture media transfected with the mutant vector. Western blot using GAPDH antibody and silver staining of the gel were provided as positive control and for normalization. The band intensities in the culture media were measured and displayed as a bar chart. ROI values are shown above the bars. Agarose gel images of PCR products are shown on the right side. W: wild-type, M: mutant.

In vitro expression assay of the wild-type and mutant ENAM tagged with EGFP demonstrated that the expression and secretion into the culture media from the mutant vector was greatly reduced compared to the wild-type (Figure 3). Protein was not detected in the cell lysate, but a reduced amount was detected in the culture media. Because the coding sequence was identical in both the wild-type and mutant vectors, it seems that the expression from the mutant vector was very low but accumulated in the culture media due to the normal signal peptide sequence. There was no significant difference in mRNA expression between the wild-type and mutant vectors.

**Figure 4:**
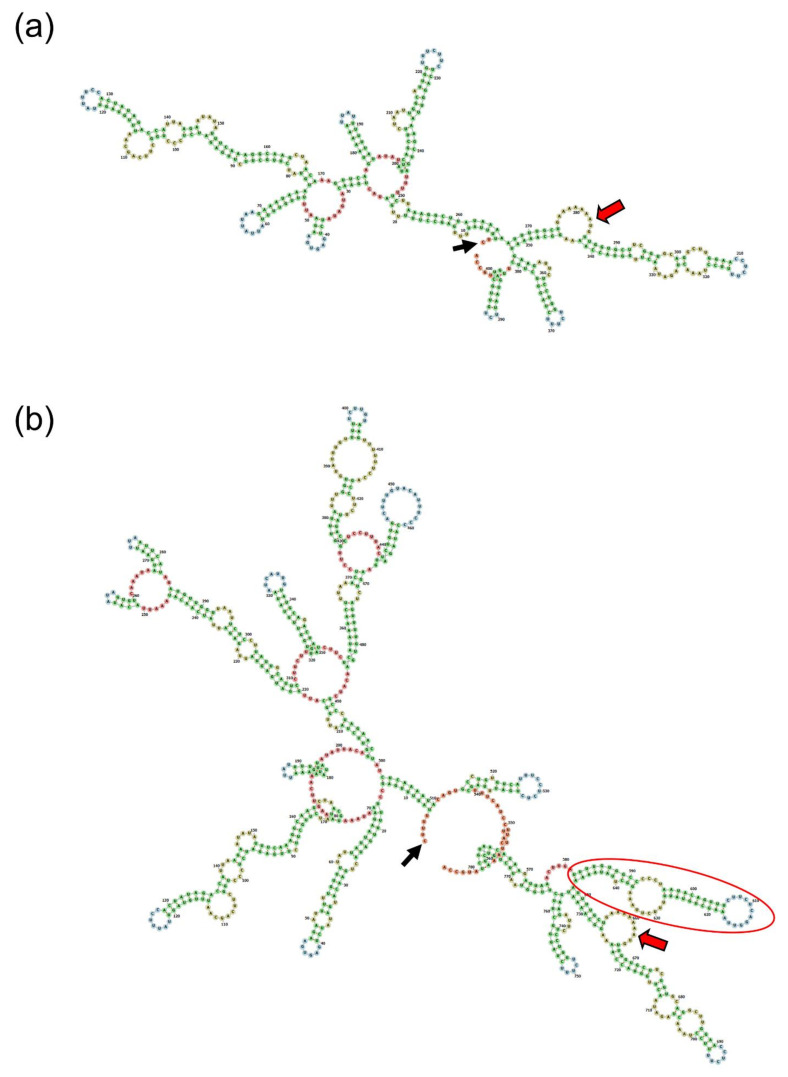
Predicted secondary structure of mRNA. (**a**) The wild-type mRNA structure. Left half is 5′ UTR, and the starting nucleotide is indicated with a black arrow. Translation initiation sequence is indicated with a red arrow. (**b**) The mutant mRNA structure. Long complex structures can be seen in the 5′ UTR, and a hairpin (red circle) is located right before the translation initiation sequence.

Prediction of the secondary structures of the wild-type and mutant mRNA sequences revealed a hairpin structure located right before the translation initiation codon, in addition to the long complex structures of the mutant mRNA (Figure 4). This hairpin structure would be one of the factors inhibiting or regulating translation.

**Figure 5:**
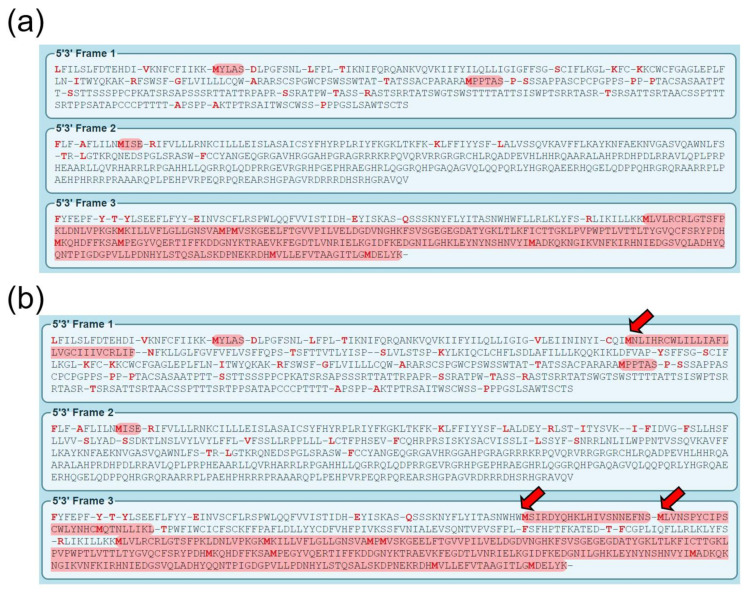
Translation prediction of the wild-type (**a**) and mutant (**b**) mRNA. Reading frame 3 is used in the wild-type. Mutant mRNA was predicted to have one out-of-frame and two in-frame upstream open reading frames (denoted by red arrows).

Predictions of the translation of the wild-type and mutant mRNA sequences showed no additional uAUG. However, they did show one out-of-frame and two in-frame uORFs in the mutant mRNA (Figure 5). Translation of the uORFs in the mutant mRNA could negatively impact the normal translation of the downstream normal ORF.

## 4. Discussion

There are three well-characterized matrix proteins: AMELX, AMBN, and ENAM. These govern structural formation of the dental enamel. ENAM content in the developing enamel matrix is estimated to be 3–5%, while AMELX is 80–90% and AMBN is roughly 5% [22]. While ENAM is the least abundant among them, it is the largest (more than 200 kDa), and is highly modified with glycosylations and phosphorylations. Once the full-length ENAM is secreted, it is cleaved from the C-terminus into several functional fragments (e.g., 32, 34 and 89 kDa fragments). ENAM is believed to be involved in enamel crystal elongation, and thus, haploinsufficiency results in hypoplastic enamel [23].

Human *ENAM* is located in chromosomal location 4q13.3 among many secretory calcium-binding phosphoprotein (SCPP) genes that are clustered in the 4q13~q21 region [24]. Human *ENAM* spans 18 kbp and is composed of ten exons. The ATG translation start codon is located in exon 3; translation ends in the last exon. Human *ENAM* encodes an 1142 amino acid full-length protein, including 39 amino acids, as the N-terminal signal peptide. Most of the peptide (946 amino acids) is encoded by the last exon.

The human *ENAM* exon 2 (61 bp) is skipped during pre-mRNA splicing. Therefore, it is not included in the mature mRNA sequence (NM_031889.3) [25]. While porcine *ENAM* mRNA (NM_214241.1) does not retain the exon 2 sequence, mouse *ENAM* exon 2 is used in the mRNA (NM_017468.3) [26,27]. It has been shown that the splice junction sequences of the acceptor and donor sites are conserved in exon 2 [28]. The functional role of this skipped exon 2, if any, is not yet known. However, the results of this study demonstrated that mutation led to the use of an alternative splicing site and this conserved exon 2 sequence.

In this study, we demonstrated that the mutation caused retention of the intron 1 and exon 2 sequences, and that the mutant mRNA with the longer 5′ UTR resulted in an attenuated translation compared to the wild-type. Even though the exact functional roles of the structural components in the mRNA (especially the 5′ UTR region) are not fully understood, it has been shown that the structures in the mRNA itself are involved in the regulation of translation [29]. Hairpin, pseudoknot, internal ribosome entry site (IRES), and highly structured 5′ UTR are examples of secondary structural elements in the 5′ UTR that can influence mRNA translation [29,30]. Prediction of the wild-type and mutant mRNA showed long complex structures in the mutant mRNA. Furthermore, a hairpin structure located right before the translation initiation sequence was predicted in the mutant mRNA. These cis-acting regulatory structures could be responsible for the attenuated translation of the mutant mRNA by restricting the binding and progressing of the translating ribosomes in the 5′ UTR.

Furthermore, it has been suggested that unstructured linear elements within 5′ UTR, such as uORFs and the sequences near the start codon of the main ORF, are likely to have a crucial impact on mRNA translation [29]. Translation prediction of the wild-type and mutant mRNA showed that the long mutant mRNA had three additional uORFs—one out-of-frame and two in-frame. These additional uORFs could be another potential contributory factor to attenuate translation of the downstream main ORF, due to inefficient translation reinitiation or a blockage to the scanning preinitiation complex due to ribosomes stalling [31].

Taken together, the reduced production of the protein caused by the mutation could be attributed to the long complex structures—and especially to the hairpin structure located right before the translation initiation codon of the mutant mRNA. However, there could be additional contributing factors, including additional uORFs. Regarding the clinical phenotype of the affected family members, the enamel defects were not severe and were clinically discernable only in limited teeth (first premolars, canines and anterior teeth). A relatively mild and random mutational effect potentially impacting transcript accessibility for translation could explain the observed mild phenotype. Furthermore, the lack of penetrance observed in the sibling warrants future investigation of modifying factor that may be at play among the reported nonpenetrating cases.

## Data Availability

The data presented in this study are openly available in ClinVar (http://www.ncbi.nlm.nih.gov/clinvar (accessed on 3 March 2021)) Submission ID: SUB9212769.

## Supplementary Materials

The following are available online at https://www.mdpi.com/article/10.3390/biomedicines9050456/s1, Table S1: Statistics for whole exome sequencing; Figure S1: DNA sequencing chromatograms of the PCR amplification products from participating individuals.
