# Supplementary material for: Translational Attenuation by an Intron Retention in the 5′ UTR of ENAM Causes Amelogenesis Imperfecta
Source: Biomedicines. 2021 Apr 22;9(5):456. doi: 10.3390/biomedicines9050456 (PMC8145330; doi:10.3390/biomedicines9050456)
Supplement: Supplementary file 1 [file biomedicines-09-00456-s001.zip › biomedicines-1169560-supplementary.pdf]

Translational Attenuation by an Intron Retention in the 5′ UTR of *ENAM* Causes Amelogenesis Imperfecta

Youn Jung Kim [1], Yejin Lee [2], Hong Zhang [3], John Timothy Wright [4], James P. Simmer [3], Jan C.-C. Hu [3] and Jung-Wook Kim [1,2*]

Table S1. Statistics for whole exome sequencing.

Sample	Total Reads	Mapping Rate (%)	Median Target Coverage	Coverage of Target Region (%)	Fraction of Target Covered with at Least	
					20×	10×
III:17	138,158,018	99.9	99	96.5	93.4	95.3
IV:4	171,693,928	99.9	113	96.5	93.6	95.4

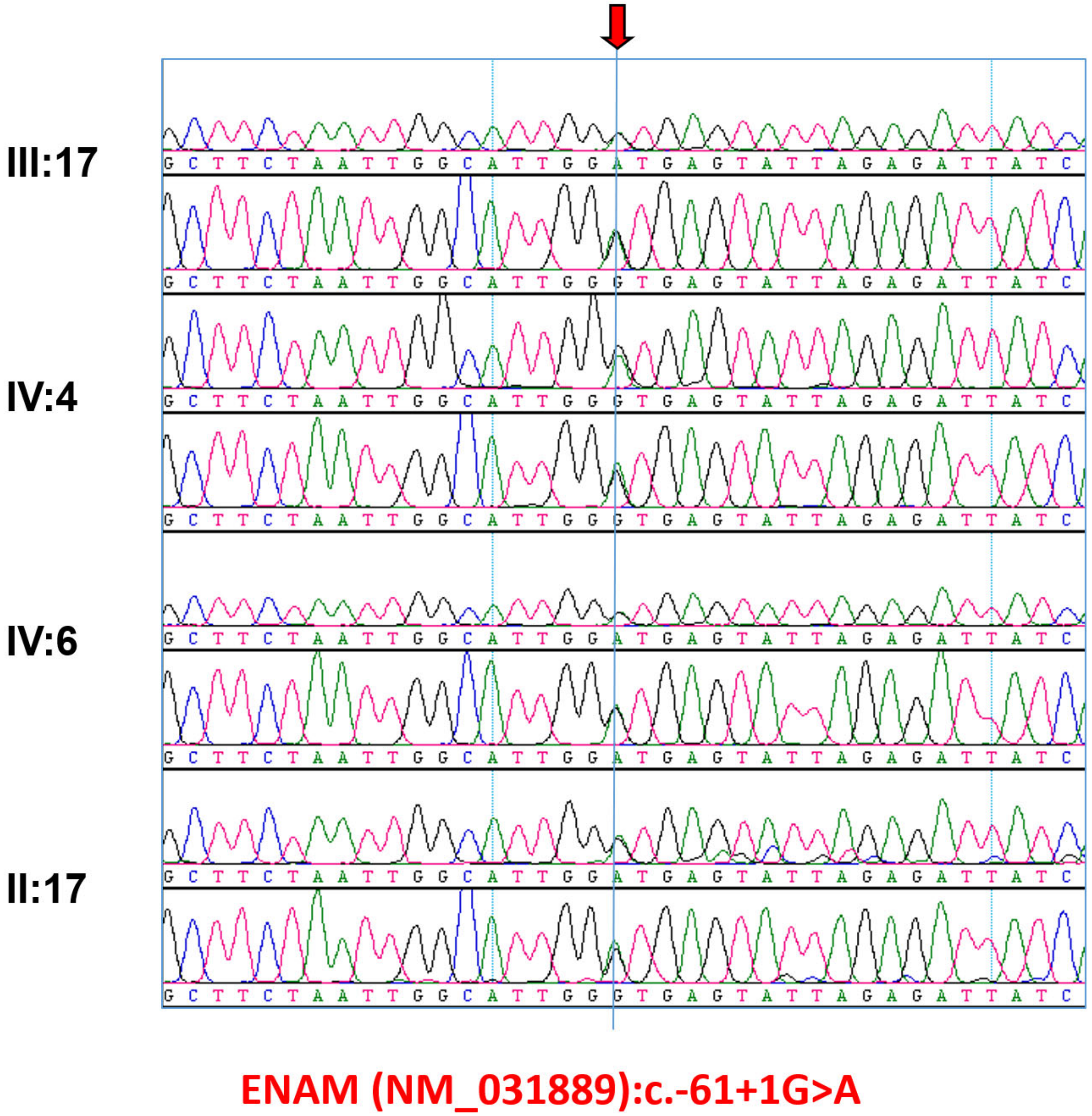

Figure S1. DNA sequencing chromatograms of the PCR amplification products from participating individuals. Individual pedigree codes are shown on the left of the chromatograms, and the mutation location is indicated with a red arrow. For each individual, forward sequencing (upper image) and reverse sequencing (lower image) chromatograms are shown.
